# Patient Satisfaction in Surgery for Parkinson’s Disease: A Systematic Review of the Literature

**DOI:** 10.7759/cureus.4316

**Published:** 2019-03-25

**Authors:** Galal A Elsayed, Joshua Y Menendez, Borna E Tabibian, Gustavo Chagoya, Nidal B Omar, Evan Zeiger, Beverly C Walters, Harrison Walker, Barton L Guthrie

**Affiliations:** 1 Neurological Surgery, University of Alabama at Birmingham, Birmingham, USA; 2 Neurological Surgery, University of Alabama at Birmimgham, Birmingham, USA; 3 Neurology, University of Alabama at Birmingham, Birmingham, USA

**Keywords:** patient satisfaction, patient reported outcome, evidence based medicine, parkinson’s disease, deep brain stimulation

## Abstract

The objective of the study was to establish how patient satisfaction with surgical treatment of Parkinson's disease (PD) has been previously measured, determine whether an ideal patient satisfaction instrument exists, and to define the dimensions of care that determine patient satisfaction with the surgical treatment of PD.

A systematic search of four online databases, unpublished sources, and citations was undertaken to identify 15 studies reporting patient satisfaction with the surgical treatment of PD. Manuscripts were reviewed and instruments were categorized by content and method axes. One study was found to utilize two distinct patient satisfaction instruments, which brought the total number of satisfaction instruments assessed to 16. Major factors influencing patient satisfaction were identified and served as a structure to define the dimensions of patient satisfaction in the surgical treatment of PD.

Studies used predominantly multidimensional (10/16), rather than global (6/16) satisfaction instruments. Generic (12/16) rather than disease-specific (4/16) instruments were utilized more frequently. Every study reported on satisfaction with outcome and four studies reported on satisfaction with outcome and care. Six dimensions of patient status, outcome and care experience affecting patient satisfaction were identified: motor function, patient-specific health characteristics, programming/long-term care, surgical considerations, device/hardware, and functional independence.

At present, no patient satisfaction instrument exists that is disease-specific and covers all dimensions of patient satisfaction in surgery for PD. For quality improvement, such a disease-specific, comprehensive patient satisfaction instrument should be designed, and, if demonstrated to be reliable and valid, widely implemented.

## Introduction and background

Patient-reported outcomes are increasingly recognized as meaningful and valid measures of successful care. This is in contrast to traditional surgeon-centered outcome parameters that include morbidity, mortality, complications, and postoperative imaging findings. Patient satisfaction is a type of patient-reported outcome distinct from reports of health, disability, and quality of life. Patient satisfaction is a measurement reflecting patients’ perception of outcome of care and has been considered for use in future reimbursement schemes. Contrary to intuition, satisfaction is not necessarily concomitant with traditional measures of surgical outcomes. There is a complex interplay between preoperative factors, the patient-physician interpersonal relationship, interactions with nurses and other hospital staff, as well as the other, more traditionally measured outcomes that determine patient satisfaction. High satisfaction leads to a high likelihood that a patient will pursue further medical care, promotes patient compliance, and has been linked to decrease the incidence of malpractice suits [1-4]. 

No consensus on patient satisfaction instruments exists in the surgical treatment of Parkinson’s disease (PD). A multidimensional, disease-specific patient satisfaction instrument could guide changes in surgical practice for quality improvement.

The goals of this review are fourfold: (1) To assess the tools that have been used to measure patient satisfaction with the surgical treatment of PD that are currently in use; (2) To determine whether a comprehensive instrument to measure patient satisfaction exists; (3) To define the dimensions of care that consistently effect patient satisfaction in the surgical treatment of PD; (4) To describe the dimensions of an ideal instrument to measure satisfaction with the surgical treatment of PD.

## Review

Methods

Search Strategy and Study Inclusion

**Figure 1:**
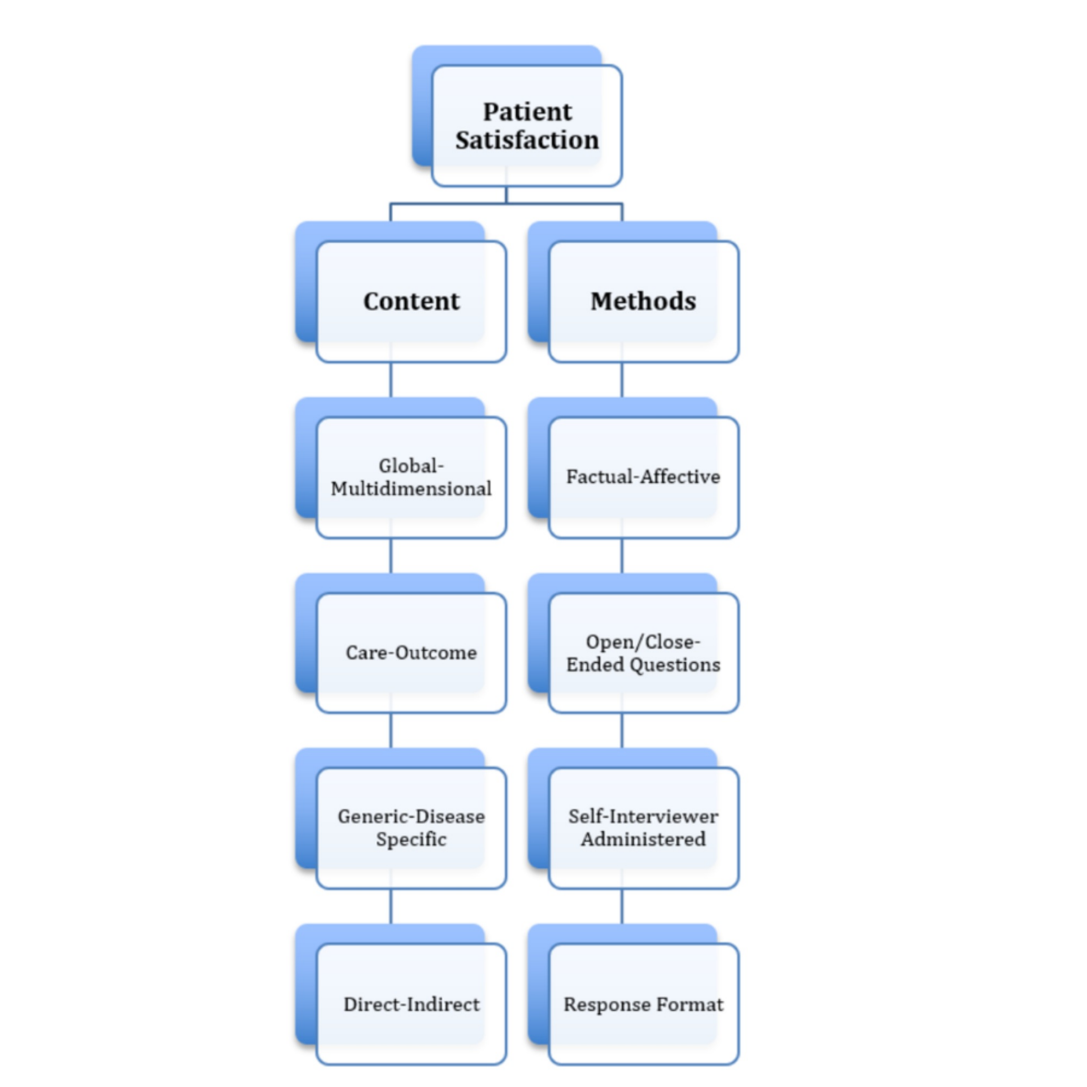
Categorization of the axes of patient satisfaction instruments

Pubmed, the Cochrane Library, CINAHL and EMBASE were systematically searched for “Patient Satisfaction” AND “Deep Brain Stimulation” AND “Parkinson’s Disease,” “Patient Expectations” AND “Deep Brain Stimulation” AND “Parkinson’s Disease,” “Physician-Patient Relationship” AND “Deep Brain Stimulation” AND “Parkinson’s Disease,” “Doctor-Patient Relationship” AND “Deep Brain Stimulation” AND “Parkinson’s Disease,” “Patient Preference” AND “Deep Brain Stimulation” AND “Parkinson’s Disease,” “Patient Satisfaction” AND “Surgery” AND “Parkinson’s Disease,” “Patient Expectations” AND “Surgery” AND “Parkinson’s Disease,” “Physician-Patient Relationship” AND “Surgery” AND “Parkinson’s Disease,” “Doctor-Patient Relationship” AND “Surgery” AND “Parkinson’s Disease,” “Patient Preference” AND “Surgery” AND “Parkinson’s Disease,” yielding 33 unique publications. Abstracts of all 33 publications were read. Manuscripts were included if they were empirical, reported measurement of patient satisfaction, and referred to the surgical treatment of PD. Manuscripts were excluded if they were review papers, if there was no measurement of patient satisfaction, or if the papers did not involve the surgical treatment of PD. If the abstract and title were not clear, then full texts were read to determine inclusion or exclusion. Using these criteria, 15 manuscripts were excluded, yielding 18 manuscripts. Then, all full-length manuscripts were read, and an additional eight manuscripts were excluded because exclusion criteria were met, yielding 10 manuscripts. Citations in all papers were reviewed, and an additional five manuscripts meeting inclusion criteria were discovered. In total this yielded 15 empirical studies measuring patient satisfaction in the surgical treatment of PD (Figure 1). 

The surgical treatment of PD was defined to include any invasive procedure for the treatment of PD, including replacement of internal pulse generators (IPG) for deep brain stimulation (DBS) arrays. Patient satisfaction was defined as any attempt of a study to gather evaluations or “affective responses” regarding a patient’s health care experience [4-5].

All manuscripts were critically read, and data were categorically extracted. Categories included: study type (retrospective, prospective, blinded, controlled, etc.), procedures performed (DBS, pallidotomy, IPG exchange, etc.), number of surgeons, length of follow-up, country of participants, number of participants, outcome measurements, patient satisfaction instrument used, intake format (self-administered or interviewer administered), validity of the instrument, and findings of the study. Siderowf et al. [6] utilized two distinct patient satisfaction measures in their study. This brought the total number of satisfaction instruments assessed in this review to 16.

From these data the characteristics of patient satisfaction instruments used in each study were classified using the criteria described by Hudak et al [4]. These authors categorized patient satisfaction instruments into two major groups: content and methods. Content describes the focus of the measurement tool, and methods describe the technique employed in the instrument. The content and methods categories each have four axes, including: global versus multidimensional questions, satisfaction with care versus satisfaction with outcome, generic questions versus disease-specific questions, and direct versus indirect questions. The four methods axes are divided into factual versus affective questions, open versus closed-ended questions, self administered versus interviewer administered instruments, and type of response format (see Figure 1). 

Through review of the findings of each study, a taxonomy of patient satisfaction with PD surgery was developed identifying the major characteristics of providers and medical care influencing patient satisfaction. In the pioneering work in elucidating the dimensions of patient satisfaction with medical care, Ware et al. formed such a taxonomy from reviewed studies to act as the basis for the development of the dimensions of care that affected patient satisfaction [5]. In a similar manner, our taxonomy acted as a structure to categorically define the dimensions of patient satisfaction in the surgical treatment PD.

Results

Current Measurement Instruments

Each patient satisfaction measurement instrument described in the 15 manuscripts were categorized using the model utilized by Hudak et al. [4] for patient satisfaction instrument content and methods. There were 16 discrete satisfaction instruments that were used since one paper described the use of two distinct instruments, 16 satisfaction instruments were assessed [6]. The content of the instruments were mostly multidimensional (62.5%) rather than global (37.5%) surveys. Each instrument measured patient satisfaction with outcome and four instruments measured satisfaction with both outcome and care (25%). Most instruments were generic and could be applied to any medical condition or intervention (75%) rather than disease-specific (25%). Each of the disease-specific instruments measured satisfaction with both outcome and care. The instruments more frequently used a purely direct (75%) rather than indirect (6.25%) approach. One instrument used in three studies utilized both direct and indirect questions (Table 1) [6-21]. 

**Table 1:** Classification of each satisfaction instrument used based on Hudak's axes Short Form-36 (SF-36), Functional Assessment of Cancer Therapy-General (FACT-G), Questions on Life Satisfaction (QLS)

Studies	Daniels et al.; Drapier et al.; Gray et al.; Siderowf et al. [6, 8-10]	D'Antonio et al [16]	Ferrara et al.; Hashimoto et al.; Kuehler et al. [11-13]	Ford et al.; Schuurman et al.; Siderowf et al.; Son et al.; Wain et al. [6, 14-15, 19, 21]	Hariz et al. [18]	Timmerman et al. [20]	Ideal
Instrument	SF-36	FACT-G	QLS	Global Satisfaction Question	Hariz instrument	Timmerman instrument	Ideal Instrument
Global or Multidimensional	Multidimensional	Multidimensional	Multidimensional	Generic	Multidimensional	Multidimensional	Multidimensional
Care or Outcome	Outcome	Outcome	Outcome and Care	Outcome	Outcome	Outcome and Care	Outcome and Care
Generic or Disease Specific	Generic	Generic	Disease Specific	Generic	Generic	Disease specific	Disease Specific
Direct or Indirect	Direct	Indirect	Direct and Indirect	Direct	Direct	Direct	Direct
Factual or Affective	Affective	Affective	Affective	Affective	Affective	Affective	Affective
Open- or Close-Ended Questions	Close-Ended	Close-Ended	Close-Ended	Close-Ended	Close-Ended	Close-Ended	Close-Ended
Interview or Questionnaire	Questionnaire	Questionnaire	Questionnaire	Questionnaire	Questionnaire	Questionnaire	Questionnaire
Response Format	Likert	Likert	Likert	Likert	Likert	Likert	Likert
Limitation of Instrument	Generic, inappropriate dimensions	Generic, inappropriate dimensions	Inappropriate dimensions	Generic, global	Generic, inappropriate dimensions	Inappropriate dimensions	None

The methods of the instruments used exclusively affective rather than factual questions. Close-ended questions rather than open-ended questions were used exclusively. Most instruments were self-administered (87.5%) rather than interviewer-administered (12.5%). The most common response format was a Likert scale (93.75%) with a varying number of gradations.

The most commonly used multidimensional patient satisfaction instrument was the Short From-36 (SF-36) instrument, which was administered in four studies [6-9]. The Questions on Life Satisfaction (QLS) scale was the next most commonly utilized multidimensional instrument used in three studies [11-13]. The QLS was also the most commonly administered disease-specific instrument and was the only instrument that measured satisfaction with regard to both outcome and care.

Dimensions of Patient Satisfaction

Of the 15 papers included in this study, three [6, 14-15] did not provide analysis that contributed to determining the dimensions of care. In these papers, the patient satisfaction instrument was used strictly to either report a level of satisfaction or compare satisfaction between procedures without analysis of which aspects of care contributed to satisfaction. Using the remaining 12 papers, [8-13, 16-21] a taxonomy of factors affecting patient satisfaction was constructed: motor function, increased off time, co-morbid conditions, dyskinesias, wound breakdown/hardware erosion, hardware failure, doctor/provider availability, ease of programming, hardware choice, surgical target, choice of surgical procedure, functional independence, patient selection, gender, and wound appearance/cosmesis. From this taxonomy, six dimensions of patient status, outcome and care experience consistently affecting patient satisfaction were identified: motor function, patient-specific health characteristics, long-term follow-up and DBS programming, surgical considerations, device/hardware, and functional independence.

Motor Function

The interaction between the improvement in motor function and patient satisfaction was explored in five studies [8-9, 11, 13, 16]. Increasing “on time,” and thus decreasing off time, motor symptoms is a major goal of therapy for PD, and surgery has been shown to be superior to medical management in achieving this goal [22]. Decreased off time was consistently linked to increased satisfaction with the surgical procedure in our review [8-9, 16]. Other components of motor function such as decreased dyskinesias, [8, 13] improved motor scores on the Unified Parkinson’s Disease Rating Scale (UPDRS), manual dexterity, ability to ambulate, and control of pharyngeal musculature that affects the ability to speak and swallow [13] were also found to be related to patient satisfaction.

Patient-Specific Characteristics

Patient-specific characteristics are intrinsic traits that cannot be modified in the acute phase prior to an operation. This includes co-morbid conditions that are not directly treated by the surgical intervention. These characteristics were found to be related to patient satisfaction in five studies [8, 12, 17-18, 20]. Specific co-morbid conditions such as depression and anxiety were tied to decreased satisfaction [8]. Misdiagnosis of a Parkinson Plus syndrome as PD also leads to decreased satisfaction, [17] and this is to be expected as the literature does not support treatment of multiple system atrophy and other Parkinson Plus syndromes with any form of surgical intervention [23-25]. 

Decreased rates of satisfaction were found to be more prevalent as patients were farther out from surgery in one study [17]. This finding is supported by the trend towards diminished function as measured by the UPDRS that has been noted in multiple studies, [26-27] but Hashimoto et al. found that satisfaction with surgical intervention remains high despite the passage of time from surgical intervention [12].

Age and gender differences were also found to affect satisfaction. Multiple studies found age to be negatively associated with patient satisfaction and one specifically found younger people to be more satisfied with a battery that could be percutaneously recharged [12, 20]. Hariz et al. [18] found that men were more satisfied with the treatment of their disease in the pre-operative period but there was no difference between genders following surgical intervention although both were more satisfied following intervention. This implies that women derive more satisfaction from the surgical intervention. 

Programming/Long-Term Care

The link between satisfaction and long-term care is clear due to the current practice of DBS being the primary surgical intervention for PD and the resultant requirement of a long-term relationship with a movement disorder specialist for programming and maintenance of the device. Farris and Giroux found that suboptimal programming of the device led to dissatisfaction early in the post-operative period [17]. Kuehler et al. highlighted the importance of the availability of the medical provider on a consistent basis to overall satisfaction with the surgical treatment of PD [13].

Surgical Considerations

Details of surgical planning and procedure, both with the intracranial portion of the process and with implantation of a pulse generator if required, were found to be linked to satisfaction in three studies [10, 17, 19].

Farris and Giroux found that intracranial leads placed in suboptimal positions led to early dissatisfaction with the surgical result [17]. The choice of a target for ablation was found to be a driver of satisfaction with one study showing that patients were more satisfied with pallidotomy than with thalamotomy. The authors of this study postulated that the difference might be due to different presenting or dominant symptoms that led to the choice of target within the deep structures of the brain. Specifically, thalamotomy was reserved for patients who presented with unilateral resting tremor as their primary and usually solitary symptom of PD. These patients did not have as debilitating a disease burden as their counterparts who underwent pallidotomy and, thus, did not derive as much benefit [10]. This finding, however, was not reproduced in other studies with more robust samples of DBS patients.

Son et al. found that patients were more satisfied with a trans-axillary, sub-pectoral implantation of a pulse generator as opposed to the standard sub-clavicular location. Patients were found to be pleased with the decreased visibility both of the pulse generator and surgical scar [19]. Kuehler et al. corroborated this point in finding that patients are more satisfied when the presence of the illness is less readily apparent [13].

Hardware

With the predominant use of DBS as surgical treatment for PD and the necessity for expensive hardware that has a finite lifespan, the presence of a linkage between hardware and patient satisfaction is not surprising. Farris and Giroux noted that hardware erosion and dysfunction are associated with dissatisfaction with surgical treatment [17]. Multiple studies have shown increased satisfaction with a rechargeable pulse generator when compared to a non-rechargeable device [20-21].

Independence

The importance of functional independence as a determinant of satisfaction was explored in three studies [11-13]. Hashimoto et al. found that both the patient’s pre-operative UPDRS ADL score, independently from change in ADL score, as well as the patient’s pre-operative required level of activity, affected satisfaction [12]. The pre-operative ADL score was found to be negatively associated with patient satisfaction, meaning that a patient who was more independent pre-operatively was more satisfied with the surgical procedure as a whole, and vice versa. The authors believe this is due to the increased expectations of recovery that might not be met in patients who have more advanced symptoms. The pre-operative required activity level was positively associated with patient satisfaction. Patients who needed to work prior to the operation were more satisfied with the operation than those who were not required to work.

Ferrara et al. and Kuehler et al. all found that functional independence and the perception of increased energy post-operatively were linked to increased patient satisfaction [11, 13].

Discussion

Patient-reported outcomes such as patient satisfaction are increasingly recognized as valid markers for therapeutic efficacy and there are discussions concerning the use of patient satisfaction in the determination of reimbursement schedules. Beyond the financial implications lie patient care issues. It is well established that patients who are more satisfied with their medical care have better long-term outcomes. Satisfied patients are more compliant with treatment regimens, develop lasting relationships with their medical providers and are more apt to present promptly with new signs and symptoms [1-2, 4, 28]. The converse is even more strongly evident with dissatisfied patients tending towards non-compliance and delayed presentation [4, 29].

Currently the field of surgical treatment for PD utilizes patient-reported outcome measures such as the UPDRS and Parkinson’s Disease Questionnaire (PDQ-39) routinely. Both of these patient-reported outcome measures fail to take into account the patients affective response to the disease process or treatment and thus do not meet the standard to be considered a patient satisfaction instrument [4]. Both of these outcome measures have been used in many of the studies cited in this review and, in several instances, were the basis of determining the domains that affect patient satisfaction with surgical treatment of PD as these outcome measures were found to be associated with either increased or decreased satisfaction through logistic regression in the individual studies. Nevertheless, these two instruments do not measure satisfaction when given in isolation.

 An ideal instrument would be a disease-specific instrument that uses a multidimensional approach to measuring satisfaction with both care and outcome. Questions should be structured in a direct manner as this is more appropriate to assess satisfaction with a specific procedure or treatment [30-31]. Questions should be affective as this allows the determination of a patient’s emotional response to a situation [32]. For ease of administration, an ideal instrument should utilize close-ended questions, a questionnaire format and a Likert response format.

The only instrument that we identified that approximates an ideal instrument is the QLS that was initially described by Kuehler et al. [13] and later utilized in other studies [11-12]. The QLS is a disease specific, multidimensional instrument that measures satisfaction with both outcome and care. The primary shortcoming of the QLS is that it does not take into account all domains that have been identified as influencing satisfaction with PD surgery. In addition, the QLS is composed of four modules that may be cumbersome to administer in a busy practice.

The absence of consideration of patient expectations is a major limitation of the QLS specifically and the current state of the measurement of patient satisfaction in the surgical treatment of PD in general. A patient’s expectations are increasingly identified as a major influence on outcomes and patient satisfaction [33-39]. Expectations, as opposed to co-morbid conditions, age, gender and other patient specific characteristics, are potentially modifiable in the short-term setting. Understanding a patient’s expectations pre-operatively can potentially aid in the appropriate selection of patients and could allow normalization of expectations, particularly through pre-operative patient education, which could lead to improved outcomes.

Limitations and Future Directions

These dimensions of care have been defined using a literature review technique. While this approach elucidates dimensions of care, there is no means of applying value to each dimension appropriately and understanding whether there are collinear dimensions that will cancel each other out. It is highly unlikely that each of these dimensions of patient satisfaction is of equal value in determining overall satisfaction. Only through design of an instrument using these dimensions and psychometric testing can appropriate values and collinear variables be established to provide utility to such a model.

## Conclusions

Patient satisfaction is increasingly understood as a distinct and important patient reported outcome measure. Despite consistent measurement of other outcome measures, including those reported by patients and observed by physicians, the current state of measurement of patient satisfaction in the surgical treatment of PD is haphazard and inconsistent. Design of an ideal instrument to measure patient satisfaction with the surgical therapy of PD would be beneficial in determining or proving the efficacy of and necessity for surgical treatment of PD.
